# Robot-assisted right hemicolectomy for ascending colon cancer with a rare common trunk for the ileocolic and middle colic arteries: a case report

**DOI:** 10.1097/RC9.0000000000000590

**Published:** 2026-06-15

**Authors:** Takashi Fujisaki, Hisaki Kato, Riku Yamamoto, Shunsuke Sakuraba, Kenichiro Tanaka, Tomoaki Ito

**Affiliations:** Department of Surgery, Juntendo University Shizuoka Hospital, Izunokuni, Shizuoka, Japan

**Keywords:** ascending colon cancer, case report, common trunk, ileocolic artery, middle colic artery, vascular anomaly

## Abstract

**Introduction:**

Anatomical variations in the arterial supply to the right colon are clinically relevant in patients undergoing laparoscopic and robotic colectomy, as misidentification of the vessels may lead to inadequate lymphadenectomy or bowel ischemia. A common trunk formed by the ileocolic artery (ICA) and middle colic artery (MCA), arising from the superior mesenteric artery (SMA), is an uncommon vascular configuration that may complicate surgical decision-making.

**Presentation of case:**

A 73-year-old man presented with constipation and abdominal pain. Colonoscopy revealed a circumferential type 2 tumor in the ascending colon, causing luminal stenosis. Histopathological examination of a biopsy confirmed moderately differentiated adenocarcinoma. Using contrast-enhanced computed tomography, wall thickening without distant metastasis was detected. Three-dimensional computed tomography angiography revealed a rare vascular anomaly in which the ICA and MCA originated from a common trunk arising from the SMA, accompanied by the absence of the right colic artery. The patient underwent an elective robot-assisted right hemicolectomy using the da Vinci Si® system. The postoperative course was uneventful; the patient was discharged on postoperative day 8. The pathological diagnosis was pT3N0M0, stage IIa adenocarcinoma. No recurrence has been observed during the 3-year follow-up.

**Discussion:**

Preoperative three-dimensional vascular imaging is useful for identifying uncommon vascular variants and translating findings into a safe intraoperative strategy. The robotic approach allows precise vascular handling in such cases.

**Conclusions:**

Awareness of such anatomical configurations may help prevent intraoperative misinterpretation and support meticulous preoperative planning, particularly in minimally invasive and robotic right colectomy.

## Introduction

In patients undergoing colorectal cancer surgery, a precise understanding of the individual mesenteric vascular anatomy is essential to determine the operative approach and achieve safe lymphadenectomy. Recent advances in three-dimensional computed tomography (3D-CT) angiography and image reconstruction techniques have enabled highly accurate and non-invasive preoperative assessment of vascular anatomy, which has become an integral part of surgical planning[1].

The branching pattern of the superior mesenteric artery (SMA) is highly variable. During D3 lymph node dissection for right-sided colon cancer, central ligation of the ileocolic artery (ICA), right colic artery (RCA), and right branch of the middle colic artery (MCA) is required^[^2,3^]^. However, due to such variations – including cases wherein these arteries share a common trunk – misidentification of the vascular anatomy during surgery may result in bowel ischemia or inadequate lymph node dissection. Therefore, accurate preoperative vascular evaluation is crucial.

HIGHLIGHTS

- Rare ileocolic artery-middle colic artery common trunk without right colic artery in ascending colon cancer
- Preoperative three-dimensional computed tomography (3D-CT) clearly identified the vascular anomaly.
- 3D-CT–guided robotic right hemicolectomy enabled safe D3 lymphadenectomy.

Although preoperative 3D-CT angiography is widely adopted in contemporary colorectal surgery, uncommon vascular configurations may still pose a substantial risk of misinterpretation during surgery, particularly for surgeons in training or in high-volume minimally invasive settings. Therefore, beyond reporting a rare anatomical variant, in the present case, we emphasize the educational value of recognizing specific vascular pitfalls and translating preoperative imaging findings into safe intraoperative decision-making.

Herein, we report a rare case of ascending colon cancer with a common trunk of the ICA and MCA arising from the SMA, successfully treated with robot-assisted right hemicolectomy, and review the relevant literature. This paper has been reported in line with the SCARE 2025 criteria[4].

## Case presentation

A 73-year-old man presented with a 1-week history of constipation and abdominal pain. The patient had no significant comorbidities or relevant past medical history. Physical examination revealed tenderness in the right lower quadrant without a palpable mass. Laboratory test results were unremarkable, including carcinoembryonic antigen (2.0 ng/mL) and carbohydrate antigen 19–9 (16 U/mL).

Colonoscopy demonstrated a circumferential type 2 lesion in the ascending colon, preventing passage of the scope. Histopathological examination of the biopsy confirmed moderately differentiated adenocarcinoma. A self-expandable metallic stent (Niti-S colonic stent) was placed for decompression, allowing for optimization and complete colonic evaluation. Computed tomography revealed an enhanced mass without lymphadenopathy or metastasis (Fig. 1a and b). 3D-CT angiography (Ziostation2®, Ziosoft, Tokyo, Japan) showed a common trunk ICA–MCA originating from the SMA, without the RCA (Fig. 1c).

**Figure 1.**
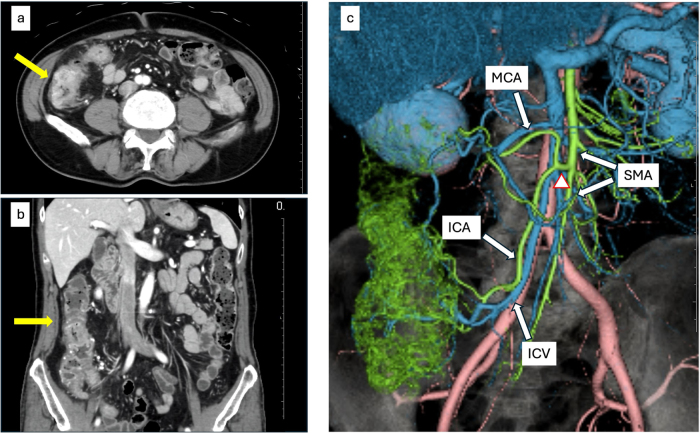
CT images. (A) Axial contrast-enhanced CT image demonstrating circumferential wall thickening of the ascending colon (arrow) without evidence of distant metastases. (B) Coronal contrast-enhanced CT image showing the extent of the ascending colon lesion (arrow). (C) Three-dimensional CT angiography revealing a common trunk of the ileocolic and middle colic arteries arising from the superior mesenteric artery (arrowhead), with the absence of the right colic artery. CT, computed tomography; SMA, superior mesenteric artery; MCA, middle colic artery; ICA, ileocolic artery; ICV, ileocolic vein

The patient was diagnosed with cT3N0M0 ascending colon cancer. A robotic approach was selected to facilitate precise vascular dissection and surgical precision.

Visualization in the presence of a complex vascular anomaly. Robotic right hemicolectomy was performed using the da Vinci Si® system. A medial-to-lateral approach was employed. The ileocolic vein was first identified and used as a landmark to expose the SMA. Careful dissection was performed along the SMA to identify the common trunk of the ICA–MCA (Fig. 2). Vascular control was achieved by individually isolating the ICA, the trunk of the MCA, and the right branch of the MCA before clipping and division. Particular attention was paid to preserving adequate perfusion to the remaining colon to avoid ischemia. The lymph node dissection was completed around the common trunk ICA–MCA, including the No. 203 and 223 stations. The ICA, ileocolic vein, and right branch of the MCA were clipped and divided. Side-to-side functional anastomosis was extracorporeally performed. The operator was a senior surgeon at our hospital. The total operative time was 5 hours and 52 minutes; the estimated blood loss was 15 mL. The postoperative course was uneventful. The final pathologically confirmed diagnosis was stage IIa adenocarcinoma of pT3N0M0 (UICC 9th edition). A total of 23 lymph nodes were harvested, with no evidence of metastasis, confirming adequate D3 lymphadenectomy. The patient declined postoperative adjuvant chemotherapy. He has been followed up at our institution with regular clinical examinations, tumor marker assessments, and imaging studies. Three years after surgery, he remains recurrence-free.

**Figure 2.**
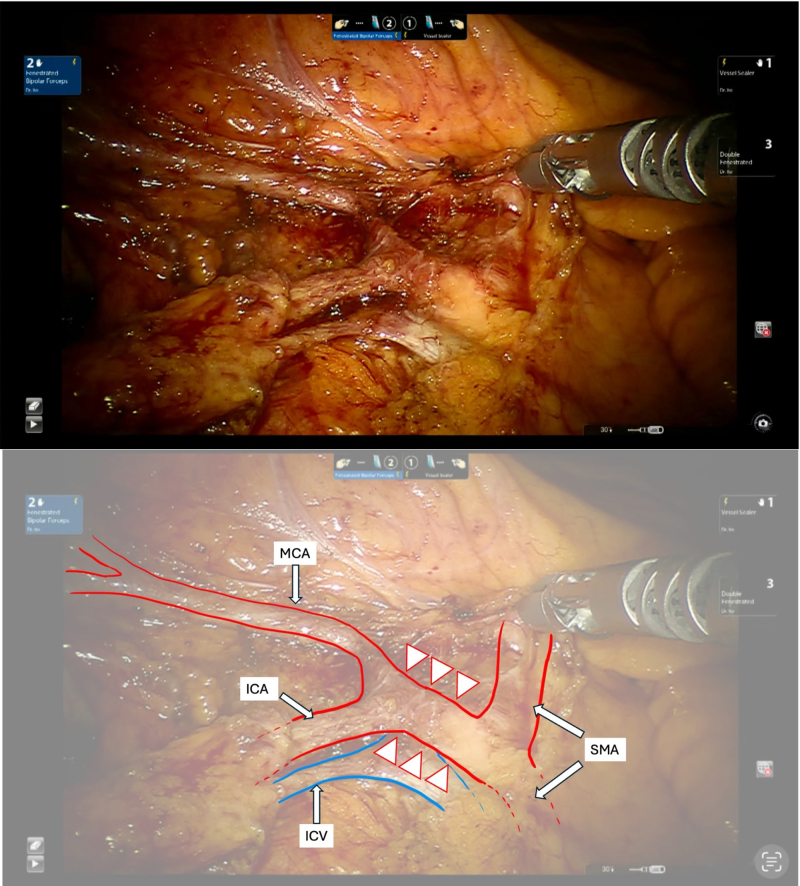
Intraoperative findings. The common trunk of the ileocolic and the middle colic arteries during robotic colectomy was identified (arrowhead). SMA, superior mesenteric artery; MCA, middle colic artery; ICA, ileocolic artery; ICV, ileocolic vein

## Discussion

The SMA branches from the abdominal aorta and supplies the small intestine and right colon. Typically, the MCA, RCA, ICA, and ileal branches arise sequentially; the MCA divides into right and left branches, supplying the hepatic and splenic flexures, respectively[2]. However, considerable variation exists in the branching patterns of the SMA. These atypical configurations can make adequate lymph node dissection technically challenging[5].

Cirocchi *et al*[6] conducted a meta-analysis of 41 studies, including 4691 patients, to clarify the vascular anatomy of the right colon for complete mesocolic excision. Among these, only four cases demonstrated a common trunk of the ICA, RCA, and MCA. A configuration in which the ICA and MCA share a common trunk and the RCA is absent, similar to that in our case, has not been described in that analysis and has been reported only in isolated case reports. Including the present case, only eight such cases of ICA–MCA common trunks have been reported to date (Table 1). Among these, five cases, based on our literature review, showed a configuration in which the MCA and ICA arose from a common trunk with the absence of the RCA^[^7–10^]^. Therefore, this common trunk configuration of the ICA–MCA is rare; accurate preoperative identification of such variants is crucial for safe surgery. Compared with previously reported cases, in which anatomical findings were mainly descriptive, surgical procedures and surgical outcomes were generally favorable; however, detailed descriptions of intraoperative vascular handling were limited. In particular, although D3 lymphadenectomy was performed in several cases, the exact strategy for identifying and managing the vascular root of the common trunk was often not clearly described. In contrast, our case provides a more comprehensive description of intraoperative management, including preoperative 3D-CT–based planning, identification of the common trunk, and a stepwise vascular control strategy during robotic surgery. Furthermore, while previous reports have primarily focused on anatomical findings, our case emphasizes the practical surgical implications of this vascular anomaly, particularly regarding safe D3 lymphadenectomy and prevention of ischemic complications. These differences highlight the added value of our report as a technical reference for surgeons encountering similar vascular variations. Although this vascular configuration cannot be regarded as exceptionally rare, its clinical relevance lies in the fact that it deviates from the anatomical patterns most commonly assumed during standard right hemicolectomy. Such deviation might not be immediately recognized intraoperatively unless it is explicitly anticipated during preoperative planning. In contrast to previous reports that primarily focused on anatomical description, the present case highlights a practical surgical perspective: namely, how preoperative recognition of an ICA–MCA common trunk directly influenced the surgical strategy, extent of lymphadenectomy, and sequence of vascular ligation during robotic surgery. This educational emphasis might be particularly valuable for surgeons less familiar with such variants.

**Table 1:** Previously reported cases and the present case of a common trunk between the ileocolic and middle colic arteries.

Case	Year	Author	Common trunk	Diagnosis	Surgical procedure	Lymph node dissection	Vascular handling detail	Postoperative complication
1	2016	Gamo *et al* 7	MCA, RCA, and ICA	Not reported	Not reported	Not reported	Not described	Not reported
2			MCA, RCA, and ICA	Not reported	Not reported	Not reported	Not described	Not reported
3	2021	Mukai *et al* 8	MCA, RCA, and ICA	Ascending colon cancer with liver metastases	Ileocecal resection and hepatectomy	D3 (common trunk of RCA and ICA)	Preoperative 3D-CT-guided strategy	Not reported
4	2022	Grytsenko *et al*^9^	MCA and ICA (absence of the RCA)	Right-sided colon cancer	Right hemicolectomy	D3	Not clearly described	Not reported
5			MCA and ICA (absence of the RCA)	Right-sided colon cancer	Right hemicolectomy	D3	Not clearly described	Not reported
6			MCA and ICA (absence of the RCA)	Right-sided colon cancer	Right hemicolectomy	D3	Not clearly described	Not reported
7	2024	Goyo *et al*^10^	MCA and ICA (absence of the RCA)	Ascending colon cancer	Laparoscopic ileocecal resection	D3 (ICA)	Use of ICG fluorescence	No complications
8	2025	Our case	MCA and ICA (absence of the RCA)	Ascending colon cancer	Robotic right hemicolectomy	D3 (ICA and right branch of MCA)	Preoperative 3D-CT-guided strategy	No complications

MCA, middle colic artery; RCA, right colic artery; ICA, ileocolic artery; ICG, indocyanine green.

In patients undergoing colorectal cancer surgery, central vascular ligation at the root of the feeding arteries, along with en bloc lymphadenectomy, is the fundamental oncological principle[11]. Regarding systematic lymph node dissection in right-sided colon cancer, root ligation of the ICA and the right branch of the MCA is required; hence, a precise understanding of the mesenteric vascular anatomy before surgery is essential for safe and adequate resection. In the context of D3 lymphadenectomy, the presence of a common trunk, as observed in our case, requires careful identification of the true vascular root to ensure oncologically adequate lymph node dissection without excessive vascular sacrifice.

Since the 2000s, laparoscopic surgery has largely replaced open surgery for colon cancer, with several large randomized controlled trials demonstrating the non-inferiority of laparoscopic colectomy in terms of survival and recurrence, as well as its superiority in postoperative recovery and minimal invasiveness^[^12–14^]^. Furthermore, recent comparative studies have shown that robotic colectomy is non-inferior or even superior to laparoscopic surgery in particular outcomes, including reduced conversion rates, decreased blood loss, and faster postoperative recovery^[^15–17^]^. The number of robotic cases is expected to continue increasing worldwide.

Therefore, a precise preoperative assessment of vascular anatomy using 3D-CT angiography is indispensable for safe and rational D3 lymphadenectomy during robot-assisted right hemicolectomy. In cases of a common trunk pattern similar to ours, misidentification of vessels can lead to bowel ischemia or anastomotic leakage. In this case, 3D-CT angiography enabled identification of the ICA–MCA common trunk. Specifically, preoperative 3D-CT allowed us to anticipate the absence of the RCA and the presence of a common trunk, which influenced the surgical plan by modifying the sequence of vascular ligation and ensuring en bloc lymphadenectomy without compromising blood supply. Hirai *et al*[18] also reported that a detailed preoperative 3D-CT evaluation of the SMA improves intraoperative safety in right colectomy. Goyo *et al*[10] demonstrated that intraoperative fluorescence of indocyanine green can facilitate safe dissection in cases of common trunks of the MCA and ICA. In our case, the da Vinci Si® system was used, which lacks Firefly™ fluorescence imaging; however, current robotic systems are equipped with this technology, and future use of indocyanine green fluorescence might further enhance intraoperative safety.

This case highlights the importance of accurately identifying vascular variants and integrating preoperative 3D-CT findings into the intraoperative strategy, particularly in robotic surgery where tactile feedback is limited. Surgeons should routinely assess vascular anatomy, plan vascular ligation in advance, and consider robotic assistance in complex cases. Although similar anatomical variations have been reported, this case demonstrates the practical value of combining 3D-CT angiography with a robotic approach to achieve safe and oncologically adequate resection.

## Conclusions

This case may not introduce a novel surgical technique or anatomical discovery; however, it provides important educational insights into the clinical implications of uncommon mesenteric vascular anatomy. By illustrating how detailed preoperative imaging can guide safe and rational intraoperative decision-making, this report serves as a practical reference for surgeons performing minimally invasive right colectomy.

## Data Availability

Not applicable.

## Research registration unique identifying number (UIN)

Not applicable. This is a single case report and does not require registration in a publicly accessible database.

## Notes

Not commissioned, externally peer-reviewed.
